# Impact of Tumor Localization and Molecular Subtypes on the Prognostic and Predictive Significance of p53 Expression in Gastric Cancer

**DOI:** 10.3390/cancers12061689

**Published:** 2020-06-25

**Authors:** Bianca Grosser, Meike Kohlruss, Julia Slotta-Huspenina, Moritz Jesinghaus, Nicole Pfarr, Katja Steiger, Alexander Novotny, Matthias M. Gaida, Thomas Schmidt, Alexander Hapfelmeier, Katja Ott, Wilko Weichert, Gisela Keller

**Affiliations:** 1 Institute of Pathology, TUM School of Medicine, Technical University of Munich, 81675 Munich, Germany; bianca.grosser@uk-augsburg.de (B.G.); meike.kohlruss@tum.de (M.K.); julia.slotta-huspenina@tum.de (J.S.-H.); moritz.jesinghaus@tum.de (M.J.); nicole.pfarr@tum.de (N.P.); katja.steiger@tum.de (K.S.); wilko.weichert@tum.de (W.W.); 2 Institute of Pathology and Molecular Diagnostics, University Hospital Augsburg, 86156 Augsburg, Germany; 3 Department of Surgery, TUM School of Medicine, Technical University of Munich, 81675 Munich, Germany; alexander.novotny@tum.de; 4 Institute of Pathology, University of Heidelberg, 69120 Heidelberg, Germany; matthias.gaida@unimedizin-mainz.de; 5 Institute of Pathology, University Medical Center Mainz, JGU-Mainz, 55131 Mainz, Germany; 6 Department of General, Visceral and Transplantation Surgery, University of Heidelberg, 69120 Heidelberg, Germany; thomas1.schmidt@med.uni-heidelberg.de; 7 Institute of Medical Informatics, Statistics and Epidemiology, Technical University of Munich, 81675 Munich, Germany; alexander.hapfelmeier@tum.de; 8 Department of Surgery, Klinikum Rosenheim, 83022 Rosenheim, Germany; katja.ott@ro-med.de; 9 German Cancer Consortium [DKTK], Partner Site Munich, Institute of Pathology, 81675 Munich, Germany

**Keywords:** p53, adenocarcinoma, gastric, gastroesophageal junction, prognosis, neoadjuvant chemotherapy, molecular subtype, microsatellite instability

## Abstract

We investigated the prognostic and predictive impact of p53 expression for gastric cancer (GC) patients treated without or with preoperative chemotherapy (CTx) and its relationship with specific molecular GC subtypes. Specimens from 694 GC patients (562 surgical resection specimens without or after CTx, 132 biopsies before CTx) were analyzed by p53 immunohistochemistry. High (H) and low (L) microsatellite instability (MSI) and Epstein–Barr virus positivity were determined previously. Our results show that aberrant p53 expression was a negative prognostic factor in uni- and multivariable analysis in the resection specimens cohort (each *p* < 0.01). Subgroup analysis showed the strongest prognostic effect for patients with distally located tumors or no CTx treatment. In the biopsy cohort before CTx, p53 did not predict response or survival. p53 expression was significantly different among the molecular subtypes in surgical resection and bioptic specimens with strong association of altered p53 with MSI-L. Patients with MSI-H and aberrant p53 showed the worst survival in the biopsy cohort. In conclusion, the prognostic impact of p53 in GC differs according to tumor localization and CTx. Altered p53 is characteristic for MSI-L, and the p53 status in biopsies before CTx delineates MSI-H subtypes with inverse prognostic impact.

## 1. Introduction

With an incidence of about one million new cases in 2018, gastric carcinoma (GC) is the sixth most common cancer worldwide, accounting for about 5.7% of all malignant diseases. The overall survival of patients with GC is limited despite the identification of specific risk factors and improved treatment concepts [1]. This highlights the need for additional molecular characterization, as well as classification, of GC to identify new therapeutic targets in different patient groups. Two of these genetic classification systems that were developed in recent years are the classification according to The Cancer Genome Atlas (TCGA) project and according to the Asian Cancer Research Group (ACRG) [2,3].

*TP53* is the most frequently mutated gene in diverse types of cancer and encodes a tumor suppressor with multiple functions related to apoptosis, cell senescence, DNA repair, cell metabolism, cell-cycle control, and grade of differentiation [4,5]. *TP53* mutations are found in about 50% of GC, and the determination of *TP53* is important for molecular tumor classification [2,3,6]. Several studies showed the negative prognostic relevance of *TP53* mutations or aberrant p53 expression in various tumor entities including GC [7,8,9,10]. However, the prognostic significance of p53 protein expression, especially in the context of perioperative chemotherapy, is controversially discussed, and no prognostic relevance of p53 was observed in some studies [9,11].

Given the fact that the predictive and prognostic role of p53 alterations in GC is still not clear, the aim of our study was to determine the impact of p53 expression in a comprehensive analysis of overall 694 carcinomas of the stomach and gastroesophageal junction including pretherapeutic biopsies of patients before preoperative chemotherapy (CTx). The inclusion of biopsies before CTx in the neoadjuvant setting also allows an exact evaluation of the predictive impact of p53 expression of responding patients with no residual tumor cells after CTx in the resected specimens. We used immunohistochemical methods to analyze p53 expression, encompassing an evaluation of p53 overexpression, as well as loss of expression, and we compared this evaluation method with next generation sequencing-based *TP53* mutation analysis in a subset of the tumors. Furthermore, we were interested in investigating p53 expression in relation to the Epstein–Barr virus (EBV) and microsatellite instability (MSI) status of the tumors, which we determined in a previous study [12].

## 2. Results

### 2.1. Study Enrolment and Patient Characteristics

**Figure 1:**
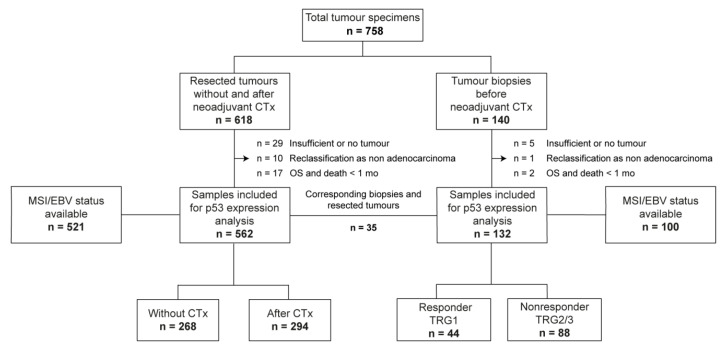
Flow chart diagram of patients and specimens inclusion. OS, Overall survival; TRG, tumor regression grade; CTx, chemotherapy; OS, overall survival; mo, months; EBV, Epstein–Barr virus; MSI, microsatellite instability.

Our study comprised a patient cohort with surgical resection specimens and a cohort with tumor biopsies before CTx. Among the 618 patients initially included in the resected cohort, 562 specimens were finally evaluable for p53 expression. Among the 140 patients initially included in the biopsy cohort before neoadjuvant CTx, 132 specimens were finally available. An overview of the study enrolment and the respective exclusion criteria are shown in Figure 1. Clinical characteristics of the patients are summarized in Table 1.

### 2.2. Frequency of Aberrant p53 Expression in the Resected Tumour Cohort

An aberrant p53 expression was found in 282 of the 562 (50.2%) resected tumors (Table 1). Among these 282 resection specimens, 211 (75%) showed an overexpression, and, in 71 (25%), a complete loss of p53 expression was observed. Examples of immunohistochemical p53 expression patterns are shown in Figure S1 (Supplementary Materials).

Among the 35 paired tumor biopsies and resection specimens, 33 (94.2%) demonstrated a concordant result, whereas, in two patients, p53 was classified as wild type in the tumor biopsy and as p53 overexpressed in the corresponding resection specimen.

Mutation analysis of the *TP53* gene by next-generation sequencing (NGS) was performed for a subset of 42 tumors, and concordant results with p53 expression analysis were demonstrated in 38 (90%) cases. Of the 22 tumors with p53 overexpression, 20 harbored missense mutations and one tumor showed an in-frame deletion mutation. Of the four cases with complete loss of p53 expression in the NGS analysis, one insertion, one nonsense mutation, and two splice variants were detected. Results are summarized in Table S2 (Supplementary Materials) and the identified mutations are listed in Table S3 (Supplementary Materials).

### 2.3. p53 Expression and Association with Patient Characteristics in the Resection Specimens Cohort

Association with clinical characteristics was performed for the 562 patients with resected tumors and revealed a significant association of p53 expression with tumor localization (*p* < 0.001) with a preponderance of an altered expression in proximally localized tumors. In addition, aberrant p53 expression was associated with male sex (*p* < 0.001), intestinal type tumors (*p* = 0.001), and a positive lymph node status (*p* = 0.007). Results are summarized in Table S4 (Supplementary Materials).

### 2.4. p53 Expression and Survival in the Resection Specimens Cohort and Specific Subgroups

Survival analyses were performed in the resection specimens and in specific subgroups stratified according to tumor localization and the presence of chemotherapy.

**Figure 2:**
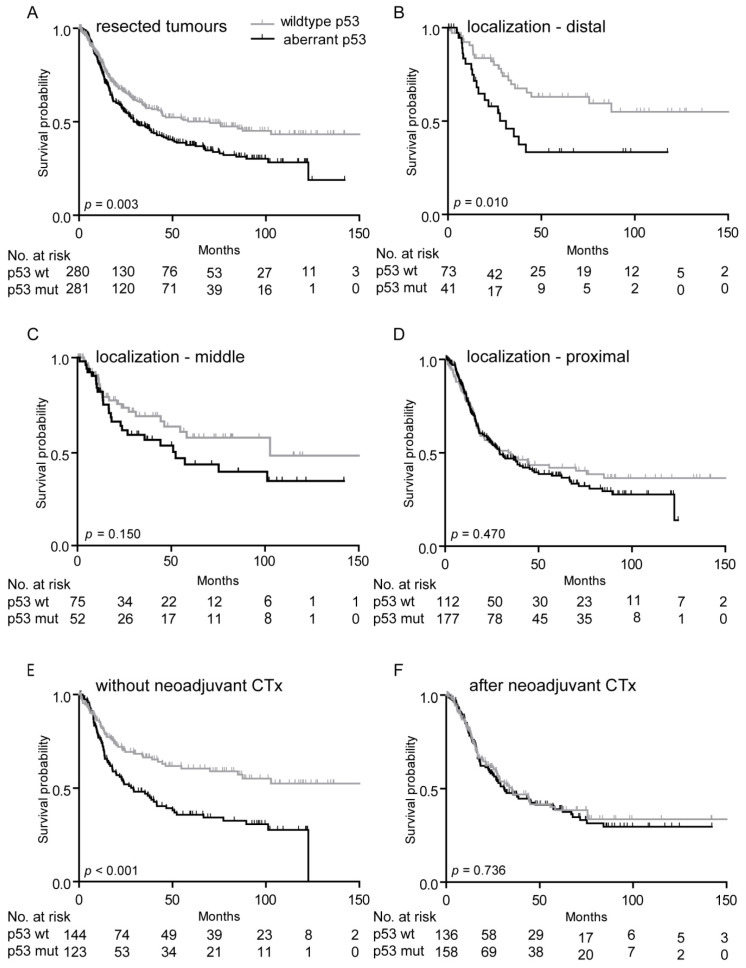
Survival of patients with resection specimens in association with p53 expression. Kaplan–Meier curves of the patients with wild-type and aberrant p53 are shown. All resection specimens (**A**); resection specimens with distal (**B**), middle (**C**), and proximal (**D**) tumor localization; resection specimens without CTx (**E**) and after neoadjuvant CTx (**F**). p53 wt, p53 wild-type expression; p53 mut, aberrant p53 expression; No., number; *p*-value of log-rank test (overall).

Aberrant p53 expression was significantly associated with worse OS in the overall resected tumor cohort (*p* log rank = 0.003; hazard ratio (HR), 1.43; 95% confidence interval (CI) 1.13–1.83) (Figure 2A, Table 2).

Subgroup analysis of OS according to tumor localization revealed a significantly worse OS of patients with aberrant p53 expression patterns especially in distally located tumors (*p* log rank = 0.010; HR, 2.19; 95% CI 1.19–4.03, Figure 2B). A tendency for a similar association was seen in tumors located in the middle third (*p* log rank = 0.150; HR, 1.51; 95% CI 0.86–2.64, Figure 2C), whereas only a slightly difference in survival was found for patients with proximal tumor localization (*p* log rank = 0.470; HR 1.13; 95% CI 0.82–1.56) (Table 2, Figure 2D).

Stratifying patients according to CTx yes or no revealed an association of aberrant p53 expression with worse prognosis in the group of patients not treated with CTx (*p* log rank < 0.001; HR, 1.95, 95% CI, 1.35–2.80, Figure 2E), but no significant difference was observed in the CTx group (*p* log rank = 0.736 HR, 1.06, 95% CI 0.76–1.47, Figure 2F). All survival data including the one-, three-, and five-year OS rates are summarized in Table 2.

Multivariable analysis was performed for the total resected cohort including clinical factors and CTx yes/no and revealed (y) pN (*p* < 0.001), M-status (*p* < 0.001), R-status (*p* < 0.001), and p53 expression (*p* = 0.006) as independent prognostic factors (Table S5, Supplementary Materials).

### 2.5. Frequency of Aberrant p53 Expression in the Pretreatment Biopsy Cohort and Response to Chemotherapy and Survival

In the pretreatment biopsy cohort, 62 of the 132 (47%) tumors showed an aberrant p53 expression with 53 of the 62 (85%) demonstrating an overexpression and nine of the 62 (15%) showing a loss of expression. Data are included in Table 1. No significant association with response to CTx was found, as 22 of the 44 (50%) responding patients and 40 of the 88 (45%) nonresponding patients showed an aberrant p53 expression (Figure S2A, Supplementary Materials). Regarding survival, patients with altered p53 showed a worse OS in the biopsy cohort, but the difference was statistically not significant (*p* log rank = 0.148, HR 1.41, 95% CI, 0.88–2.26). Data are included in Table 2, and the survival curve is shown in Figure S2B (Supplementary Materials). Analysis of OS in subgroups stratified according to tumor localization in the biopsy cohort also revealed a significant association of aberrant p53 with decreased OS only in distally (*p* log rank = 0.04) located tumors but not in those located in the middle (*p* log rank = 0.47) and proximal (*p* log rank = 0.58) third (data not shown).

### 2.6. p53 Expression and Correlation with the EBV- and MSI-Positive Molecular Subgroups and Survival

In a previous study we determined the EBV and MSI status of our tumors by classifying MSI in high (MSI-H) and low-MSI (MSI-L) [12]. Complete information of p53 expression and the EBV and MSI status was available for 521 resection specimens (244 primary resected and 277 after CTx) and for 100 tumor biopsies before CTx (Figure 1).

**Figure 3:**
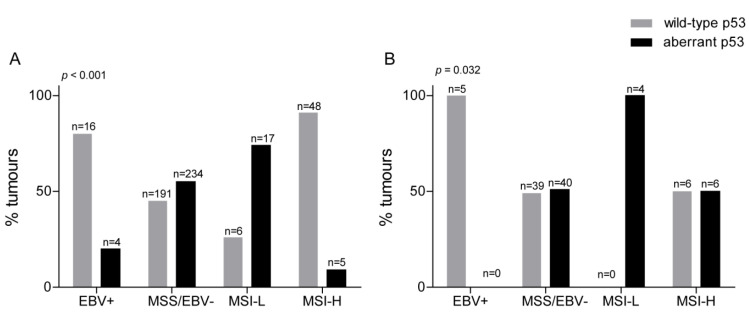
Association of p53 expression with MSS/EBV−, MSI-L, MSI-H, and EBV+ tumor subtypes. The comparison of the four molecular subgroups with p53 expression is shown in the resection specimens (**A**) and the biopsy cohort before CTx (**B**). EBV(+), Epstein–Barr virus-positive; EBV(−), Epstein–Barr virus-negative; MSI-L, low microsatellite instability; MSS, microsatellite stable; MSI-H, high microsatellite instability; *p*-value of chi-squared test.

Firstly, we now asked if p53 expression varied among these specific molecular subtypes. Our results showed that the distribution of altered p53 expression among the EBV, MSI-H, MSI-L, and microsatellite stable (MSS) subgroups was significantly different in both the resected and the biopsy cohort (*p* < 0.001 and *p* = 0.032 respectively) (Figure 3A,B). The most obvious features were an association of wild-type p53 with EBV positivity (+) and of aberrant p53 expression with the MSI-L phenotype in both tumor cohorts. In addition, an association of wild-type p53 expression with the MSI-H phenotype was found in the resected tumors, whereas, in the biopsy cohort, the number of MSI-H tumors with wild-type or aberrant p53 expression was balanced.

Secondly, we were interested if p53 expression might modulate a particular prognostic effect of the molecular subgroups, which we showed in our previous study [12]. As, in the biopsy cohort, only tumors with both wild-type and aberrant p53 expression were present in the MSS/EBV− and the MSI-H group, we restricted this analysis to these two patient subgroups.

**Figure 4:**
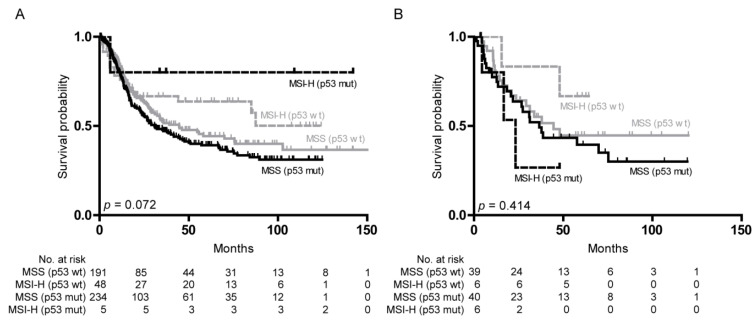
Survival of the patients with MSI-H and MSS/EBV− tumors and association with p53 expression. Kaplan–Meier curves of the patients with MSI-H and MSS/EBV− tumors and association with p53 expression in resection specimens (**A**) and biopsies before CTx (**B**). EBV(+), Epstein–Barr virus-positive; EBV(−), Epstein–Barr virus-negative; MSI-L, low microsatellite instability; MSS, microsatellite stable; MSI-H, high microsatellite instability; p53 wt, p53 wild-type expression; p53 mut, aberrant p53 expression; No., number; *p*-value of log-rank test (overall).

Regarding survival in the resected specimens cohort, MSI-H was associated with better OS in the subgroups with aberrant and wild-type p53 expression compared to the respective MSS subgroups (median MSI-H not reached in each group and MSS/EBV− 44.6 and 30.9 months in the wild-type and aberrant p53 expression group, overall *p* log rank = 0.072). In the tumor biopsy cohort before CTx, patients with MSI-H tumors showed a better OS compared to the MSS/EBV− tumors only in the p53 wild-type group (median MSI-H not reached, MSS/EBV− 44.6 months), whereas an even worse OS for MSI-H compared to MSS/EBV− tumors was observed in the presence of aberrant p53 expression (median MSI-H 23.4 months, MSS/EBV− 36.6 months, overall *p* log rank = 0.414). Survival curves are shown in Figure 4, and data are summarized in Table S6 (Supplementary Materials).

## 3. Discussion

In this study, we performed a comprehensive analysis of p53 expression and its prognostic and predictive impact in two GC patient cohorts encompassing tumor biopsies before treatment and resected tumor specimens without and after preoperative CTx. We identified an aberrant p53 expression in 50% and 47% in the resected and biopsy cohort, respectively, which is similar to other studies reporting an altered p53 expression or *TP53* mutations in the range of 38–59% [2,13,14,15,16,17,18].

An aberrant p53 expression was associated with worse survival of patients in the resected tumor cohort in our study. This is in line with several reports on a negative prognostic role of p53 in GC and esophageal adenocarcinomas, although other studies did not find a correlation of altered p53 with survival in this tumor entity [9,11,14,19,20]. Analyzing the prognostic relevance of p53 in different patient subgroups, we showed—to the best of our knowledge—for the first time that the prognostic role of p53 depends on the localization of the tumor within the stomach with an association of aberrant p53 expression with worse OS in tumors located in the distal and middle third but to a much lesser extent in proximally located tumors. Our results may contribute to some clarification of controversial results regarding p53 and prognosis in GC as they clearly demonstrate that the prognostic significance depends on specific clinical characteristics of the analyzed tumor cohort. In addition, we found a significantly higher frequency of aberrant p53 expression in proximally located tumors, which is in line with other reports [21]. Thus, taken together, this suggests a variation of p53 function in proximally and distally located tumors and underlines the molecular heterogeneity of GC and differences in cancer biology of tumors in various anatomic locations within the same organ.

In our study, a negative prognostic impact of aberrant p53 expression was only seen in the subgroup of patients with resected tumors not treated with preoperative CTx, whereas, in the CTx group, there was no difference. One could speculate that this difference might be related to an alteration of p53 expression by the applied neoadjuvant CTx. However, we did not find significant differences in the expression patterns comparing corresponding tumor biopsies and resection specimens. As patients with resected tumors after CTx had more frequently proximally located tumors and more advanced tumor stages than patients in the group not treated with CTx, the diverse survival rates may be due to this discrepancy of the CTx and non-CTx patient group and, thus, may also reflect a variation of the prognostic effect of p53 in the different anatomic localizations of the tumor. Furthermore, an association of altered p53 with good response or increased survival after CTx in particular in East Asian GC patients was described [22,23], and it cannot be completely excluded that altered p53 might have contributed to a somewhat increased chemotherapy sensitivity and better survival in at least some of our patients.

In our analysis of the biopsy cohort before CTx treatment, we did not find an association with response to therapy in terms of tumor regression or with respect to the survival of the patients. This is in line with a report analyzing only a small number of pretherapeutic tumor biopsies [24], whereas other studies reported that mutated or aberrant expression of p53 indicated chemoresistance [25,26,27]. These inconsistent results may be due to various reasons related to the complexity and functional diversity of p53 and p53 mutations, as well as to differences in the applied chemotherapeutic regimens and in patient characteristics, while methodical aspects of p53 determination most likely play a role.

Regarding the association of the aberrant p53 expression with the molecular subtypes, we found significant differences. One of the most striking observations was the significant association of altered p53 expression with the MSI-L phenotype, which supports the consideration of MSI-L as a specific type of microsatellite instability and as an own molecular subtype. In contrast, the EBV-positive group and, preferentially, the MSI-H group were associated with wild-type p53. These results are essentially similar to the TCGA study with only 3.8% *TP53* mutations in EBV-positive tumors and 59.1% and 39.1% in MSI-L and MSI-H tumors, respectively, when analyzing the TCGA data using the cBioPortal for Cancer Genomics (www.cbioportal.org) [2]. Regarding a possible effect of altered p53 expression on survival of the patients in the specific molecular subgroups, we observed an interesting difference in the MSI-H group, particularly in the biopsy cohort. In this cohort, patients with MSI-H and aberrant p53 expression had the worst survival, whereas the MSI-H phenotype with wild-type p53 showed the best survival compared to the MSS/EBV-negative group, which showed no obvious difference if p53 was aberrant or not. We are aware that the small number of tumors limits the interpretation and our findings have to be confirmed in further studies. Nevertheless, our results may shed some interesting aspect on the recent discussion that neoadjuvant chemotherapy may even harm patients with MSI-H tumors [28,29,30]. Multiple functions of p53 related to apoptosis, regulation of cell cycle, and DNA repair were described. However, results of in vivo short hairpin RNA (shRNA) screens targeting p53-regulated genes suggest that, in particular, the coordination of DNA repair processes seems to be most important [31], which may be specifically relevant in an MSI-H tumor before chemotherapeutic treatment. Our finding may also support the notion that MSI-H is not a homogeneous subgroup. MSI-H subtypes which differ in their molecular profiles and the extent of global tumor mutation burden were described [32], and, in particular, for GC, different MSI-H subtypes in the context of anatomical location or histopathology were demonstrated [33]. Furthermore, for colorectal cancer, it was shown that the prognostic relevance of *TP53* may be mediated by a subtype specific effect on the tumor immune cell infiltration with *TP53* mutations identifying a non-immunogenic subgroup [34], and one can speculate that this may interfere with the immunogenic properties of the MSI-H phenotype in a particular subset of the patients.

Our study has limitations. The most relevant limitation refers to its retrospective nature and, thus, our study has to be considered an explorative analysis. Furthermore, despite the relatively overall high number of analyzed cases, the evaluation of effects of altered p53 expression in specific subgroups leads to an inevitable small number of patients, which underlines the explorative and observational characteristic of our study and warrants further investigations. Specifically, due to the complexity of p53 function and the diverse effects of p53 mutations, detailed functional experiments are necessary to clarify the role of p53 in the context of response to chemotherapy and in the background of the different molecular subtypes, particularly regarding the MSI-H subtype.

A strength of our study encompasses the large size of our patient population, as well as the immunohistochemical analysis, which included the evaluation of loss of expression and not only an evaluation of p53 overexpression, which in addition showed a high concordance with NGS-based *TP53* mutation analysis in a subset of the tumors. Furthermore, our analysis of paired biopsies and resected tumors demonstrated that a reliable evaluation of p53 expression in tumor biopsies is possible and valid.

## 4. Materials and Methods

### 4.1. Patients

Surgical resection specimens from 618 patients with adenocarcinomas of the stomach and the gastroesophageal junction that were treated between 2001 and 2013 at the Department of Surgery of the University of Heidelberg and between 2001 and 2012 at the Department of Surgery of the Technical University Munich were initially included in the study. Tumors of 56 patients were excluded and immunohistochemical analysis of p53 was evaluable for 562 resected tumors (294 after neoadjuvant CTx and 268 without CTx) (Figure 1).

Diagnostic pretherapeutic tumor biopsies before CTx of 140 patients treated at the Department of Surgery at the Technical University of Munich between 1993 and 2013 were initially evaluated for inclusion in the study, and 132 of them were selected for final evaluation (Figure 1).

Paired tumor biopsies and corresponding resection specimens were present in 35 patients. Tumor tissues of a subset of the patients were already included in a recent study on the prognostic and predictive role of EBV positivity and high and low MSI in GC [12]. An overview of the enrolment of the patients for the present study is shown in Figure 1, and the clinical characteristics of the patients evaluated for p53 are summarized in Table 1.

### 4.2. Ethics Statement

The study was approved by the institutional Ethics Review Boards at the Technical University of Munich (reference: 502/15s) and at the University of Heidelberg (reference: 301/2001), and all the patients agreed at the beginning of their treatment that their tissue may be used for research purposes. This study was performed in accordance with the Declaration of Helsinki.

### 4.3. Chemotherapy and Surgery

Patients were treated with platinum/5-fluorouracil (5FU) based chemotherapeutic regimens as detailed in Table S1 (Supplementary Materials). Radical oncological surgery included a D2 lymph-adenectomy as described in detail previously [12].

### 4.4. Response Evaluation

Response to preoperative CTx was determined histopathologically and was classified into three tumor regression grades (TRG): TRG1, TRG2, and TRG3, which corresponded to <10%, 10–50%, and >50% residual tumor cells/tumor bed respectively [35]. In the tumor biopsy cohort, all three tumor regression grades were represented. Patients with TRG1 were classified as responders, while those with TRG2 and TRG3 were classified as nonresponders.

In the resected tumor cohort after CTx, patients with TRG1 were not included either due to the complete absence of tumor cells or very low tumor cell content, which impeded molecular analysis.

### 4.5. Follow-Up and Overall Survival

Follow-up was performed as described previously [36], and overall survival (OS) was defined as the time between the date of operation and death by any cause.

### 4.6. Tissue Microarray Construction

Formalin-fixed paraffin-embedded (FFPE) tumor samples were assembled into a tissue microarray (TMA) using a Tissue Microarrayer (Beecher Instruments, Sun Praierie, WI, USA) with a core size of 0.6 mm. A minimum of three tumor cores from the tumor invasion front and tumor center were taken from the primary tumors in areas previously marked by one pathologist.

### 4.7. Immunohistochemistry

Immunohistochemistry was performed on 2-μm sections from each TMA using a p53 primary antibody (DAKO, clone DO-7) at a dilution of 1:200. Stainings were run on an automated immunostainer with an iVIEW DAB detection kit (Ventana Medical Systems, Roche, Mannheim, Germany). Stained slides were digitalized using a high-throughput scanning system (AT2, Leica, Wetzlar, Germany), and evaluation was performed with Aperio eSlide Manager. Immunohistochemical expression of p53 in tumor cells was assessed by a three-tiered classification scheme as proposed by Köbel et al. [37] and Darb-Esfahani et al. [38] in ovarian and breast carcinoma, respectively. p53 wild-type (WT) expression was defined as less than 60% tumor cells displaying nuclear p53 staining with variable intensity. An aberrant p53 expression was present as either a complete loss of expression with 0% nuclear staining of tumor cells or an overexpression of p53 with more than 60% nuclear staining of tumor cells with medium to high intensity [37,38].

### 4.8. DNA Isolation, Analysis for MSI, and Detection of EBV

DNA isolation, analysis for MSI using the five microsatellite markers (BAT25, BAT26, D2S123, D5S346, D17S250) as recommended by the National Cancer Institute, and detection of EBV were performed as described recently [12]. According to their MSI and EBV status, the tumors were classified in four molecular subgroups, EBV (+), MSI-H (high: ≥2/5 unstable markers), MSI-L (low: 1/5 unstable marker), and microsatellite stable (MSS)/EBV-negative (−).

### 4.9. TP53 Mutation Analysis by Next-Generation Sequencing (NGS)

Mutation analysis of the *TP53* gene was performed by targeted sequencing using the Ion Torrent platform (Thermo Fisher Scientific, Waltham, MA, USA) and a custom-designed sequencing gene panel (Ion AmpliSeq, Thermo Fisher Scientific) encompassing the coding exons of the *TP53* gene. The multiplex PCR based Ion AmpliSeq targeted sequencing technology (Thermo Fisher Scientific) was used for DNA library preparation, and amplification of target regions was performed using the Ion AmpliSeq Library Kit v2.0, as well as the specific primer panel as described previously [39]. The preparation of the DNA libraries is described in more detail in the Supplementary Materials (Supplementary Methods). Automated template preparation of the final libraries, as well as chip loading (Ion 520, 530, or 540), was performed on an Ion Chef instrument, and sequencing was done using an Ion S5XL instrument (Thermo Fisher Scientific). Data analysis was performed referring to Pfarr et al. 2017 [39], and ANNOVAR was used to annotate the sequence variants [40].

### 4.10. Statistical Analysis

Chi-squared tests were used for hypothesis testing of differences between the relative frequencies. Kaplan–Meier estimates of survival rates were compared by log rank tests. Relative risks were estimated by hazard ratios (HRs) from univariable Cox proportional hazard models. A multivariable Cox proportional hazards model was built by stepwise forward variable selection using likelihood-ratio tests of pre-therapeutically and post-therapeutically available clinical factors. Statistical analyses were performed using SPSS, Version 25 (IBM Corp., Armonk, NY, USA). Exploratory 5% significance levels (two-tailed) were used for hypothesis testing.

## 5. Conclusions

In conclusion, our study shows that the prognostic impact of p53 in GC differs according to tumor localization and treatment with chemotherapy, arguing for diverse roles of p53 in a specific cellular context even in the same organ. While p53 represents an independent prognostic factor for specific subgroups, it showed no predictive relevance for chemotherapy response. Regarding the relationship with specific molecular subtypes, our results indicate that MSI is not a homogeneous subgroup. Firstly, because of the obvious association of p53 with MSI-L tumors, this supports the consideration of MSI-L as a separate subgroup. Secondly, because of the results in the biopsy cohort, this indicates that the p53 status may delineate two MSI-H subtypes with inverse prognostic impact for patients treated with preoperative CTx. Further studies are required to confirm and elucidate these findings in more detail.

## Figures and Tables

**Table 1:** Patient characteristics.

Resection Specimens							Tumor Biopsies		
		All		Without Neoadjuvant CTx		After Neoadjuvant CTx		Before Neoadjuvant CTx	
Category	Value	*n*	%	*n*	%	*n*	%	n	%
**Cases**	Total	562 ^1^	100	268	100	294	100	132	100
**Age**	Median	64.6		68.5		60.9		63.4	
	Range	28.3–90.9		32.1–90.9		28.3–81.2		39.5–79.1	
**Follow-up period** **(mo)**	Median	61.6		68.9		54.0		61.8	
	95% CI	53.1–70.1		56.6–81.2		44.1–63.9		60.1–63.5	
**Overall survival** **(mo)**	Median	41.6		51.0		32.4		48.0 ^2^	
	95% CI	30.5–52.7		20.4–81.6		22.4–42.4		30.0–66.0	
**Sex**	Male	409	72.8	179	66.8	230	78.2	99	75.0
	Female	153	27.2	89	33.2	64	21.8	33	25.0
**Localization**	Proximal	289	51.4	103	38.4	186	63.3	93	69.7
	Middle	127	22.6	65	24.3	62	21.1	23	17.4
	Distal	114	20.3	81	30.2	33	11.2	15	11.4
	Total/linitis	28	5.0	15	5.6	13	4.4	2	1.5
	n/a	4	0.7	4	1.5	0	0	0	0
**Laurén histological subtype**	Intestinal	317	56.4	144	53.7	173	58.8	68	51.5
	Non intestinal	245	43.6	124	46.3	121	41.2	64	48.5
**Tumor grade**	G1/2	111	19.8	66	24.6	45	15.3	32	24.2
	G3/4	388	69.0	201	75.0	187	63.6	100	75.8
	n/a	63	11.2	1	0.4	62	21.1	0	0
**cT**	cT2	129	23.0	116	43.3	13	4.4	8	6.1
	cT3/cT4	431	76.6	151	56.3	280	95.2	107	81.0
	n/a	2	0.4	1	0.4	1	0.4	17	12.9
**(y) pT ^3^**	(y) pT0	0	0	0	0	0	0	8	6.1
	(y) pT1	52	9.3	37	13.8	15	5.1	12	9.1
	(y) pT2	68	12.1	40	14.9	28	9.5	19	14.4
	(y) pT3	298	53.0	129	48.1	169	57.5	71	53.8
	(y) pT4	144	25.6	62	23.1	82	27.9	21	15.9
	n/a	0	0	0	0	0	0	1	0.8
**(y) pN ^3^**	Negative	173	30.8	95	35.4	78	26.5	58	43.9
	Positive	389	69.2	173	64.6	216	73.5	73	55.3
	n/a	0	0	0	0	0	0	1	0.8
**Metastasis status**	No	483	85.9	247	92.2	236	80.3	95	72.0
	Yes	79	14.1	21	7.8	58	19.7	32	24.2
	n/a	0	0	0	0	0	0	5	3.8
**Resection status**	R0	433	77.0	218	81.3	215	73.1	113	85.6
	R1	129	23.0	50	18.7	79	26.9	18	13.6
	n/a	0	0	0	0	0	0	1	0.8
**p53 expression**	Wild-type	280	49.8	144	53.7	136	46.3	70	53.0
	Aberrant	282	50.2	124	46.3	158	53.7	62	47.0
**EBV status**	Positive	20	3.6	6	2.2	14	4.8	95	72.0
	Negative n/a	501 41	89.1 7.3	238 24	88.8 9.0	263 17	89.5 5.8	6 31	4.5 23.5
**MSI status**	MSS	445	79.2	203	75.7	242	82.3	84	63.6
	MSI-L	23	4.1	11	4.1	12	4.1	5	3.8
	MSI-H n/a	53 41	9.4 7.3	30 24	11.2 9.0	23 17	7.8 5.8	12 31	9.1 23.5

CTx, preoperative chemotherapy; CI, confidence interval; EBV, Epstein–Barr virus; MSS, microsatellite stable; MSI-L, low microsatellite instability; MSI-H, high microsatellite instability; n/a, no data available. ^1^ One tumor without survival data. ^2^ OS was defined as time between the date of operation and death by any cause. For two patients who were not operated, the date of start of CTx was used. ^3^ Classification according to 7th Edition UICC 2007.

**Table 2:** Survival data of the patient cohorts and subgroups in association with the p53 expression.

Patient Cohorts and Subgroups	p53 Expression	No.	Events	Survival Probability (%)			Median Survival (mo)	HR ^1^	*p*-Value ^1^
				1 yr	3 yrs	5 yrs	(95% CI)	(95% CI)	
Resection specimens total cases	Wild-type	280	115	82.5	57.3	50.2	70.0 (35.7–104.3)	1 ref.	0.004
	Aberrant	281	154	77.9	47.1	37.6	29.4 (21.1–37.7)	1.43 (1.13–1.83)	
	Total ^2^	561	269	79.8	51.8	34.4	41.6 (30.5–52.7)		
Resection specimens proximal localization	Wild-type	112	59	77.4	47.6	42.1	33.9 (14.5–53.3)	1 ref.	0.471
	Aberrant	177	100	77.7	47.0	37.8	29.1 (19.2–39.0)	1.13 (0.82–1.56)	
	Total	289	159	77.6	47.2	39.4	29.4 (20.9–37.9)		
Resection specimens middle localization	Wild-type	75	23	82.5	66.1	47.9	102.7 (n.a.)	1 ref.	0.154
	Aberrant	52	26	77.4	53.2	39.3	51.0 (25.7–76.3)	1.51 (0.86–2.64)	
	Total	127	49	82.2	61.8	48.8	75.3 (36.7–113.9)		
Resection specimens distal localization	Wild-type	73	22	90.7	67.5	63.0	nr	1 ref.	0.012
	Aberrant	41	20	80.6	41.8	33.4	31.0 (15.7–46.3)	2.19 (1.19–4.03)	
	Total	114	42	87.0	58.5	52.6	87.5 (n.a.)		
Resection specimens without neoadjuvant CTx	Wild-type	144	50	82.2	66.4	60.5	nr	1 ref.	<0.001
	Aberrant	123	72	74.2	46.0	35.8	29.1 (14.8–43.4)	1.95 (1.35–2.80)	
	Total	267	122	78.9	57.3	48.6	51.0 (20.4–81.6)		
Resection specimens after neoadjuvant CTx	Wild-type	136	65	81.1	47.0	38.6	35.1 (21.7–48.5)	1 ref.	0.736
	Aberrant	158	82	79.9	46.8	39.1	31.0 (21.2–40.8)	1.06 (0.76–1.47)	
	Total	294	147	80.5	46.9	38.9	32.4 (22.4–42.4)		
Tumour biopsies before neoadjuvant CTx	Wild-type	70	34	86.8	61.6	45.9	57.1 (37.5–76.7)	1 ref.	0.148
	Aberrant	62	36	76.5	50.5	41.5	36.6 (23.2–50.0)	1.41 (0.88–2.26)	
	Total	132	70	82.0	56.5	43.7	48.0 (30.0–66.0)		

Ref, reference; nr, not reached; HR, hazard ratio; CI, confidence interval. ^1^ *p*-value and HR based on Cox proportional hazards model. ^2^ For one of the 562 analyzed patients, no survival data were available.

## Supplementary Materials

The following are available online at https://www.mdpi.com/2072-6694/12/6/1689/s1. Table S1. Chemotherapy regimens of the preoperatively treated patients; Table S2. Comparison of immunohistochemical p53 expression analysis and NGS based TP53 mutation analysis of 42 gastric carcinomas; Table S3. List of TP53 mutations identified by NGS in gastric carcinomas; Table S4. p53 expression of resection specimens and association with patient characteristics; Table S5. Multivariable analysis of survival including p53 expression and clinical factors in the resection specimens; Table S6. Survival data of the patient cohorts in association with the MSI status and the p53 expression; Figure S1. Examples of immunohistochemically p53 expression; Figure S2. p53 expression in the tumor biopsies before CTx and association with response and survival.
