# Supplementary material for: Impact of Tumor Localization and Molecular Subtypes on the Prognostic and Predictive Significance of p53 Expression in Gastric Cancer
Source: Cancers (Basel). 2020 Jun 25;12(6):1689. doi: 10.3390/cancers12061689 (PMC7352381; doi:10.3390/cancers12061689)
Supplement: Supplementary file 1 [file cancers-12-01689-s001.pdf]

cancers MDPI

Supplementary Materials

Impact of Tumour Localization and Molecular Subtypes on the Prognostic and Predictive Significance of p53 Expression in Gastric Cancer

Bianca Grosser, Meike Kohlruss, Julia Slotta-Huspenina, Moritz Jesinghaus, Nicole Pfarr, Katja Steiger, Alexander Novotny, Matthias M. Gaida, Thomas Schmidt, Alexander Hapfelmeier, Katja Ott, Wilko Weichert and Gisela Keller

Supplementary Methods

Massive Parallel Sequencing Using a Gastric Cancer Specific Sequencing Panel

DNA library preparation was performed using the Ion AmpliSeq targeted sequencing technology (Thermo Fisher Scientific, Waltham, MA, USA) and the custom designed gastric cancer (GC) sequencing panel consisting of four primer pools. The concentration and quality of amplifiable DNA was quantified previously by qPCR for each sample. For library preparation with a 4-pool primer panel, a separate PCR preparation mix was applied for each pool. 2.5 ng or 3.75 ng DNA (depends on determined DNA concentration by qPCR) was mixed with 2.5 µL of the respective 2x concentrated primer pool and 1 µL Ion AmpliSeq HiFi Master Mix for each pool. The amplification conditions in a thermal cycler were as follows: initial step at 99 °C for 2 min, followed by 21 cycles with a denaturation step at 99 °C for 15 sec and annealing/elongation at 60 °C for 4 min.

After amplification, the four single PCR reactions from each sample, which correspond to one tumour sample, were combined to one single reaction mix respectively for further processing and partially digested by FuPa reagent followed by barcode and adapter ligation (Ion Xpress Barcode Adapters, Thermo Fisher Scientific). The barcoded libraries were purified using AMPure XP magnetic beads (Beckman Coulter, Krefeld, Germany) and quantified by qPCR using the Ion Library Quantitation Kit (Thermo Fisher Scientific). DNA libraries with concentrations >25 pM were further processed for sequencing as previously described [1]. For automated template preparation, the libraries were diluted to a final concentration of 25 pM and libraries were pooled and processed using the Ion 510/520/530 or the Ion 540 chef Kit on an Ion Chef instrument. Sequencing was performed using the S5 chemistry on an Ion S5XL instrument (Thermo Fisher Scientific).

Supplementary Tables

Table S1. Chemotherapy regimens of the preoperatively treated patients.

Preoperative Chemotherapy Regimens	Tumour Biopsies Before CTx		Resected Tumours After CTx	
	n	%	*n*	%
Total	132	100	294	100
Cis + 5-FU or Cap	108	81.8	122	41.5
Ox + 5-FU or Cap	19	14.4	42	14.3
Cis + 5-FU + Doc or Pac	2	1.5	25	8.5
Ox + 5-FU + Doc	0	0	23	7.8
Cis or Ox + 5-FU or Cap + Epi	2	1.5	59	20.1
Others	1	0.8	22	7.5
n/a	0	0	1	0.3

Cis, cisplatin; Ox, oxaliplatin; 5-FU, 5-fluorouracil; Cap, capecitabine; Doc, docetaxel; Pac, paclitaxel; Epi, epirubicin; Others, combination of Cis/Ox with other agents or cross over between different treatment regimens; n/a, no data available.

Table S2. Comparison of immunohistochemical p53 expression analysis and NGS-based *TP53* mutation analysis of 42 gastric carcinomas.

IHC	Number of Tumours	NGS	Number of Tumours	Concordant Results	Discrepant Results
**p53 overexpression**	22	Missense mutation	20 [1]	21	
		Non-frameshift deletion	1		
		No *TP53* mutation	1		1
**Loss of p53 expression**	4	Truncating mutation [2]	4	4	
**WT**	16	No *TP53* mutation	13	13	
		Missense mutation	2		3
		Splice Variant	1		
**Total number of analysed tumours**	42		42	38	4

IHC, Immunohistochemistry; NGS, Next-generation sequencing; WT, wild-type. [1] One tumour harboured both a *TP53* missense and nonsense mutation; [2] Truncating mutations include splice variants, a nonsense mutation and a frameshift insertion.

Table S3. List of *TP53* mutations identified by NGS in gastric carcinomas.

cDNA [1] Description	Protein Description	Exon	Type of Mutation	Number of Tumours Harbouring Mutation
c.338dupT	p.F113fs	4	Frameshift insertion	1
c.541C>T	p.R181C	5	Missense	1
c.404G>A	p.C135Y	5	Missense	1
c.524G>A	p.R175H	5	Missense	2
c.392A>T	p.N131I	5	Missense	1
c.527G>A	p.C176Y	5	Missense	1
c.659A>G	p.Y220C	6	Missense	1
c.560_562-7del	X187_splice	6	Splice variant	1
c.722C>T	p.S241F	7	Missense	1
c.743G>A	p.R248Q	7	Missense	1
c.764_766del	p.I255_256del	7	Non-frameshift deletion	1
c.673-1G>C	X225_splice	7	Splice variant	1
c.817C>T	p.R273C	8	Missense	4
c.844C>T	p.R282W	8	Missense	5
c.818G>A/T	p.R273H/L	8	Missense	2
c.824G>A	p.C275Y	8	Missense	1
c.892G>T	p.E298*	8	Nonsense	1
c.916C>T	p.R306*	8	Nonsense	1
c.920-2A>G	X307_splice	9	Splice variant	1

[1] RefSeq Number: NM_000546.

Table S4. p53 expression of resection specimens and association with patient's characteristics.

Category	Value	p53 Expression		
		Wild-Type (n)	Aberrant (n)	*p*-value [1]
**Cases**	Total	280	282	
**Age [years]**	Median	64.4	64.6	
	Range	31.7–85.5	28.3–90.9	
**Age Median**	<Median	141	139	0.800
	≥Median	139	143	
**Sex**	Male	185	224	<0.001
	Female	95	58	
**Localization**	Proximal	112	177	<0.001
	Middle	75	52	
	Distal	73	41	
	Total	17	11	
	n/a	3	1	
**Laurén histological subtype**	Intestinal	139	178	0.001
	Non intestinal	141	104	
**Tumour grade**	G1/2	52	59	0.495
	G3/4	196	192	
	n/a	32	31	
**cT**	cT2	258	254	0.156
	cT3/4	22	26	
	n/a	0	2	
**(y) pT**[2]	(y) pT1/2	65	55	0.283
	(y) pT3/4	215	227	
**(y) pN**	Negative	101	72	0.007
	Positive	179	210	
**Metastasis status**	No	248	235	0.074
	Yes	32	47	
**Resection status**	R0	219	214	0.512
	R1	61	68	
**Neoadjuvant chemotherapy**	No	144	124	0.077
	Yes	136	158	

n, number of cases; n/a, no data available. [1] *p*-value of Chi-Squared Test; [2]Classification according to 7th Edition UICC 2007.

Table S5. Multivariable analysis of survival including p53 expression and clinical factors in the resection specimens.

Variables [1]	HR	95% CI	*p*-value [2]
**All resected specimens**			
**(y) pN status**			
pN0	1	-	-
pN1	2.96	2.12–4.14	<0.001
**M status**			
M0	1	-	-
M1	1.82	1.32–2.50	<0.001
**Resection status**			
R0	1	-	-
R1	1.96	1.49–2.58	<0.001
**p53 expression**			
Wild-type	1	-	-
Aberrant	1.40	1.10–1.79	0.006

HR, Hazard Ratio; CI, confidence interval; 1 ref., reference. [1] Included factors: age, sex, localization, Laurén subtypes, pT, pN, M-status, R-status, CTx (yes/no), p53 expression. [2] *p*-value based on forward likelihood ratio Cox's regression model.

Table S6. Survival data of the patient cohorts in association with the MSI status and the p53 expression.

	MSI Status	No.	Events	Survival Probability [%]			Median Survival [mo]	HR[1]	*p*-value [1]
				1 yr	3 yrs	5 yrs	(95% CI)	(95% CI)	
**Wild-type p53 expression**									
Tumour biopsies before neoadjuvant CTx	MSS/EBV^{-}	39	19	78.2	55.1	44.7	44.6 (21.1–68.1)	1 ref.	0.313
	MSI-H	6	2	100	83.3	66.7	nr	0.47 (0.11–2.03)	
	Total	45	21	81.3	59.2	47.9	48.1 (n.a.)		
Resection specimens (total)	MSS/EBV^{-}	191	85	83.1	51.6	44.3	44.6 (25.9–63.3)	1 ref.	0.199
	MSI-H	48	18	75.9	66.6	63.7	nr	0.72 (0.43–1.19)	
	Total	239	103	81.6	55.0	48.8	55.7 (22.7–88.7)		
**Aberrant p53 expression**									
Tumour biopsies before neoadjuvant CTx	MSS/EBV^{-}	40	24	77.3	52.1	39.5	36.6 (23.9–49.3)	1 ref.	0.479
	MSI-H	6	3	80.0	26.7	26.7	23.4 (7.7–39.1)	1.55 (0.46–5.25)	
	Total	46	27	77.7	49.7	38.1	31.3 (18.2–44.5)		
Resection specimens (total)	MSS/EBV^{-}	234	125	77.9	47.1	39.2	30.9 (20.2–41.6)	1 ref.	0.179
	MSI-H	5	1	80.0	80.0	80.0	nr	0.26 (0.04–1.86)	
	Total	239	126	78.0	47.9	40.1	31.4 (21.0–41.8)		

Ref, reference; nr, not reached; HR, Hazard Ratio; CI, confidence interval. [1] *p*-value and HR based on of Cox proportional-hazards model.

Supplementary Figures

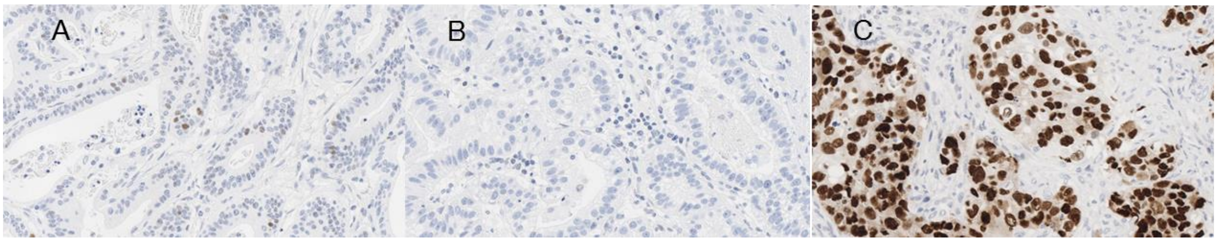

Figure S1. Examples of immunohistochemically p53 expression. Wild-type p53 expression with <60% nuclear expression with variable intensity (WT) (**A**), complete loss of expression (CA) (**B**) and overexpression with ≥60% nuclear expression with mediate to strong intensity in tumour cells (OE) (**C**).

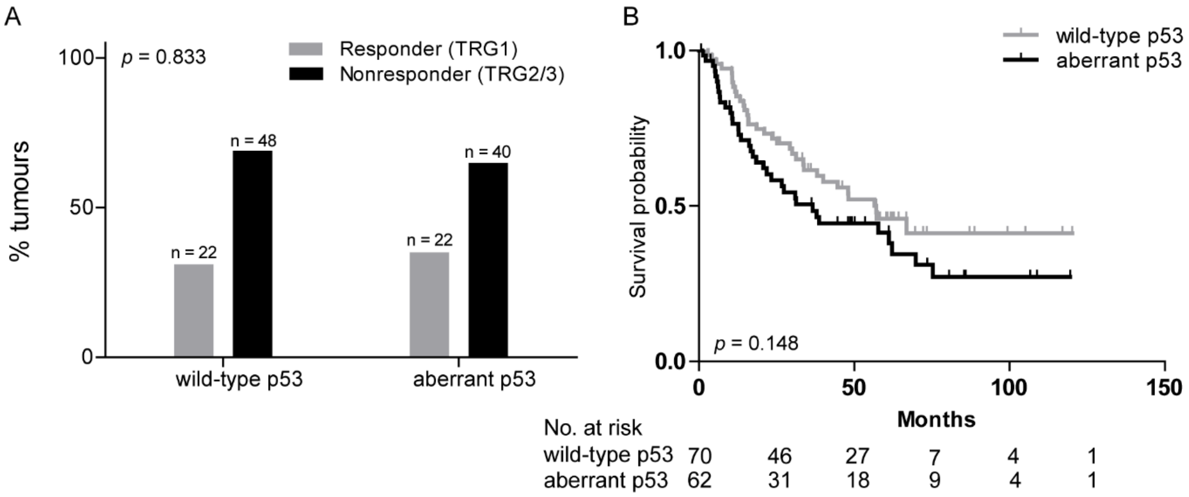

Figure S2. p53 expression in the tumour biopsies before CTx and association with response and survival. Association of p53 expression and response (**A**) and Kaplan-Meier curves (**B**) of the patients with wild-type and aberrant p53 in tumour biopsies before CTx are shown. p53 wt, p53 wild-type expression; p53 mut, aberrant p53 expression; No., number; TRG, tumour regression grade; *p*-value of Chi-Squared test (**A**); *p*-value of log-rank test (overall) (**B**).

Supplementary Reference

1. Pfarr, N.; Darb-Esfahani, S.; Leichsenring, J.; Taube, E.; Boxberg, M.; Braicu, I.; Jesinghaus, M.; Penzel, R.; Endris, V.; Noske, A.; et al. Mutational profiles of Brenner tumors show distinctive features uncoupling urothelial carcinomas and ovarian carcinoma with transitional cell histology. *Genes Chromosomes Cancer.* **2017**, *56*, 758–766, doi:10.1002/gcc.22480.
